# Persistent epigenetic reprogramming of sweet taste by diet

**DOI:** 10.1126/sciadv.abc8492

**Published:** 2020-11-11

**Authors:** Anoumid Vaziri, Morteza Khabiri, Brendan T. Genaw, Christina E. May, Peter L. Freddolino, Monica Dus

**Affiliations:** 1 The Molecular, Cellular and Developmental Biology Graduate Program, The University of Michigan, Ann Arbor, MI 49109, USA.; 2 Department of Molecular, Cellular and Developmental Biology, College of Literature, Science, and the Arts, The University of Michigan, Ann Arbor, MI 49109, USA.; 3 Department of Biological Chemistry, The University of Michigan, Ann Arbor, MI 48109, USA.; 4 Program in Biology, College of Literature, Science, and the Arts, The University of Michigan, Ann Arbor, MI, 48109, USA.; 5 The Neuroscience Graduate Program, The University of Michigan, Ann Arbor, MI 49109, USA.; 6 Department of Computational Medicine and Bioinformatics, The University of Michigan, Ann Arbor, MI 48109, USA.

## Abstract

Diets rich in sugar, salt, and fat alter taste perception and food preference, contributing to obesity and metabolic disorders, but the molecular mechanisms through which this occurs are unknown. Here, we show that in response to a high sugar diet, the epigenetic regulator Polycomb Repressive Complex 2.1 (PRC2.1) persistently reprograms the sensory neurons of *Drosophila melanogaster* flies to reduce sweet sensation and promote obesity. In animals fed high sugar, the binding of PRC2.1 to the chromatin of the sweet gustatory neurons is redistributed to repress a developmental transcriptional network that modulates the responsiveness of these cells to sweet stimuli, reducing sweet sensation. Half of these transcriptional changes persist despite returning the animals to a control diet, causing a permanent decrease in sweet taste. Our results uncover a new epigenetic mechanism that, in response to the dietary environment, regulates neural plasticity and feeding behavior to promote obesity.

## Teaser

Diets high in sugar persistently alter sensory responses to sweetness through the actions of the PRC2 complex to promote obesity.

## INTRODUCTION

Diets high in processed foods promote higher calorie intake and weight gain, increasing the risk for chronic and metabolic diseases (*1*). How these foods cause overconsumption, however, is still unclear. Processed foods are high in salt and fat, which we are genetically programmed to like because of their high caloric density (*2*). Evidence is emerging that the levels of salt, sugar, and fat in diets can alter taste sensation in humans (*3*–*5*), raising the question of whether these sensory changes may influence food intake, obesity, and metabolic disease (*6*, *7*). This idea is supported by a number of recent animal studies that found changes in taste, neural responses, and food preferences in rodents fed high-nutrient diets (*8*–*13*). However, because of the complexity of the mammalian taste system and the lack of genetic tools, we know next to nothing about the molecular mechanisms through which diet composition affects taste sensation and obesity. Thus, studies in genetically tractable model organisms could help shed light on this question and define evidence-based strategies to curb the prevalence of obesity and metabolic disease, which currently affects billions of people worldwide.

We recently found that high dietary sugar dulls the responses of the *Drosophila melanogaster* taste neurons to sweet stimuli, causing higher food intake and weight gain, arguing that the effects of diet on taste are conserved (*14*, *15*). Here, we exploited the exquisite genetics tools of the fly and the relative simplicity of its sensory system to uncover the mechanisms through which high levels of dietary sugar reshape the sensory neurons to promote weight gain and obesity. We report that the Polycomb Repressive Complex 2.1 (PRC2.1), a chromatin-silencing complex conserved from plants to humans (*16*), tunes the activity of the sweet sensory neurons and taste sensation in response to the food environment by repressing a neurodevelopmental transcriptional program that shapes the synaptic, signaling, and metabolic properties of these cells. This diet-dependent transcriptional remodeling persisted even when animals were returned to the control diet, leading to lasting changes in sweet taste behavior that depended on the constitutive activity of PRC2.1. Together, our findings suggest that diet composition activates epigenetic mechanisms that reprogram sensory responses to food; this sensory reprogramming determines the perception of future stimuli, leading to long-lasting alterations in behavior that increase the risk for obesity and metabolic disease.

## RESULTS

### PRC2.1 modulates sweet taste in response to diet

*D. melanogaster* flies fed high dietary sugar experience lower sweet taste sensation as a result of the decreased responsiveness of the sweet sensory neurons to sugar stimuli (*14*, *15*). Given the importance of sensory cues to control eating and recent data that diet also affects taste in mammals (*8*–*12*), we set out to identify the molecular mechanisms through which dietary experience shapes sensory responses. We previously reported that sweet taste deficits develop within 2 to 3 days upon exposure to the high sugar diet, depended on the concentration of sugar in the diet, but were independent of fat accumulation and weight gain (*14*). We, thus, reasoned that gene regulatory mechanisms may be involved in modulating the responses of the sensory neurons to diet composition. To test this hypothesis, we conducted a screen for gene regulatory and epigenetic factors necessary for the sweet taste defects caused by a high sugar diet. To do this, we fed control (*w1118^CS^*) and mutant flies a control diet (CD; ~5% sucrose) or a diet supplemented with 30% sucrose [sugar diet (SD)] for 7 days and then tested their taste responses to sweetness using the proboscis extension response (PER) (*17*). This behavioral assay measures taste perception by quantifying the amount of proboscis extension (0 = no extension, 0.5 = partial extension, and 1 = full extension) when the fly labellum—where the dendrites and cell bodies of the taste neurons are located (Fig. 1A)—is stimulated with three different concentrations of sucrose (30, 10, and 5%); when used in this way, PER generates a taste curve where flies respond more intensely to higher sugar stimuli (Fig. 1B, gray circles). Flies fed a sugar diet show a marked decrease in PER to sucrose compared to control diet flies (Fig. 1B, gray squares); however, mutants for the core PRC2—which includes the histone 3 lysine 27 (H3K27) methyltransferase *enhancer of zeste* [*E*(*z*)] and the obligate accessory factors *suppressor of zeste 12* [*Su*(*z*)*12*] and *extra sex combs* (*esc*) (Fig. 1B)—had largely the same PER on a control and sugar diet (Fig. 1B, right, red shades). Notably, the PER of *Su*(*z*)*12* mutant flies on a CD was lower than control animals, likely because of the additional roles this gene plays in heterochromatin formation (*18*). To confirm the role of PRC2 in sweet taste deficits, we supplemented the control and sugar diet with EED226, a PRC2 inhibitor (herein referred to as EEDi) that destabilizes the core complex by binding to the trimethyl H3K27 (H3K27me3) binding pocket of EED (the homolog of *esc* in *Mus musculus*) (*19*). While animals fed a SD+ vehicle [10% dimethyl sulfoxide (DMSO)] experienced lower PER, those fed an SD+ EEDi retained normal sweet taste responses in a dose-dependent manner (Fig. 1C), consistent with results from the PRC2 mutants. Thus, mutations and inhibition of PRC2 prevent the blunting of sweet taste that occurs in the high sugar food environment.

**Fig. 1:** PRC2.1 modulates sweet taste in response to diet. (**A**) Schematic of sweet sensory neurons. (**B**) The PRC2 complex: *E*(*z*), *Su*(*z*)*12*, *esc*, and the accessory protein *Pcl*. (B to **G**) Taste responses (*y* axis) to stimulation of the labellum with 30, 10, and 5% sucrose (*x* axis) of age-matched male (B) *w1118^cs^*, *E*(*z*)*^c249/+^*, *Su*(*z*)*12^c253/+^*, and *esc^c289/+^* flies on a control or sugar diet. *n* = 34 to 68. *w1118^cs^* on a CD compared to *E*(*z*)*^c249/+^* (ns), *Su*(*z*)*12^c253/+^* (****), and *esc^c289/+^* (ns). (C) *w1118^cs^* flies on a control or sugar diet with vehicle (10% DMSO) or 5 and 8 μM EEDi. *n* = 32 to 43. (D) *w1118^cs^*, *Pcl^c429/+^*, and *Pcl^T1/+^* flies on a control or sugar diet. *n* = 36 to 82. (E) *Gr5a > Pcl^RNAi-1^* and transgenic controls. *n* = 42 to 63. (F) *Gr5a > Pcl* and transgenic controls on a control diet. *n* = 36 to 61. (G) *Gr5a > Pcl* flies on a control diet with vehicle (10% DMSO) or 8 μM EEDi. *n* = 30 to 35. In all panels, flies were on a control (circle) or sugar (square) diet for 7 days. Data are shown as means ± SEM. ****P < 0.0001, ***P < 0.001, **P < 0.01, and *P < 0.05.

In flies, PRC2 forms two main complexes, PRC2.1 and PRC2.2, which contain distinct accessory factors that influence the targeting of the core complex to the chromatin (*18*). Mutations in the *Polycomb-like* (*Pcl*) gene, the accessory factor to PRC2.1, phenocopied PRC2 mutants and prevented sweet taste deficits in flies fed a sugar diet (Fig. 1D). In contrast, flies with deficits in the PRC2.2-members *Jumonji*, *AT-rich interactive domain 2* (*Jarid2*) and *jing* still showed a blunting of sweet taste responses in flies fed a sugar diet (fig. S1A). Members of the PRC1 and the recruiter complex Pho RC were also not required for the taste changes in responses to a sugar diet (fig. S1, B to D). Thus, the PRC2.1 complex is necessary for the sensory changes that occur in the high sugar environment.

We next asked whether PRC2.1 is required specifically in the sweet sensory neurons to decrease their responses to sweet stimuli on the sugar diet. To do this, we used the GAL4/UAS system to knock down *Pcl* in neurons that express the sweet taste receptor gene *Gustatory receptor 5a* with the *Gr5a-GAL4*, which labels ~60 cells in the proboscis of adult flies (*20*); we selected *Pcl* to narrow the effect to the PRC2.1 complex. Incidentally, *Gr5a^+^* cells also respond to fatty acids (*21*), but this modality was not affected by the high sugar diet (*14*). Knockdown of *Pcl* in *Gr5a^+^* neurons using two independent RNA interference (RNAi) transgenes (50% knockdown efficiency; fig. S2A) prevented sweet taste deficits in animals fed a sugar diet (Fig. 1E and fig. S2B). *Pcl* knockdown, however, had no effect on sweet taste on a control diet (fig. S2C), in accordance with the observation that *E*(*z*) and *Pcl* mutants have normal sweet taste on a control diet (Fig. 1, B to D) and suggesting that these phenotypes are uncovered only by the high sugar food environment.

Because *Pcl* is thought to target the PRC2 core complex to chromatin (*18*), we hypothesized that its overexpression may be sufficient to induce sweet taste deficits even in the absence of a high sugar food environment. Overexpression of *Pcl* specifically in the *Gr5a^+^* neurons lead to sweet taste deficits in flies fed a control diet compared to transgenic controls (Fig. 1F). The effects of *Pcl* overexpression were abolished by treatment with the PRC2 inhibitor EEDi (Fig. 1G), arguing that *Pcl* overexpression causes sweet taste deficits entirely through the action of PRC2 and not through some yet unidentified mechanism. *Pcl* overexpression had no effect on the number of *Gr5a^+^* neurons in the proboscis (fig. S2D), and so, the taste deficits cannot be attributed to a decrease in the number of cells. To exclude the possibility that the effects of manipulating *Pcl* on sweet taste were developmental, we used the temperature-sensitive *tubulin-GAL80^ts^* transgene to limit expression of *UAS-Pcl* and *Pcl^RNAi^* only to adult flies. Switching the flies to the nonpermissive temperature and the respective diet 4 days after eclosion resulted in the same effects on sweet taste as using the *Gr5a-GAL4* alone (fig. S2E). Together, these experiments establish that the PRC2.1 complex is required cell-autonomously in the *Gr5a^+^* neurons to mediate the effects of a high sugar diet on sweet taste.

### *Pcl* mutant animals have the same sensory responses to sucrose, regardless of diet

Flies on a high sugar diet have lower sweet taste because the neural responses of the taste neurons to sweet stimuli are decreased (*14*, *15*). Since *Pcl* mutants have identical sweet taste sensation on a control and sugar diet (Fig. 1), we hypothesized that the responses of the sensory neurons to sucrose stimulation should also be similar. To test this, we expressed the genetically encoded presynaptic calcium indicator *UAS*-*GCaMP6s-Brp-mCherry* (*22*) in the sweet-sensing neurons and measured their in vivo responses to stimulation of the proboscis with 30% sucrose in control and *Pcl* mutant animal brains (Fig. 2, A to D). As we previously showed, the responses to sucrose stimulation were lower in control flies fed a high sugar diet (Fig. 2, A and B, and fig. S2F); however, in *Pcl* mutants, the magnitude of calcium responses to sucrose was nearly identical between animals fed a control diet and sugar diet (Fig. 2, C and D, and fig. S2G), matching the behavioral data (Fig. 1); this rescue was not due to an increase in the number of sweet taste cells (Fig. 2E). Despite the fact that the *Pcl* mutant or *Pcl* knockdown animals had identical PER to control flies on a control diet (Fig. 1 and fig. S2), the calcium responses to sucrose in the taste neurons were lower in the mutants.

**Fig. 2:** *Pcl* mutant animals have the same neural responses to sucrose, regardless of diet. (**A**) Setup for in vivo calcium imaging: The proboscis is stimulated with 30% sucrose while recording from the SEZ containing the presynaptic terminals of the sweet taste neurons here (labeled with *synaptotagmin::GFP*). (A and **C**) Average %Δ*F*/*F*_0_ calcium traces to stimulation of the proboscis (arrow) in age-matched male *Gr64f > GCaMP6s-Bruchpilot-mCherry* (A) and *Gr64f > GCaMP6s-Bruchpilot-mCherry*;*Pcl^c429^* flies (C). (**B** and **D**) peak %Δ*F*/*F*_0_ responses on a control or sugar diet for flies in (A) and (C), respectively. *n* = 28 to 46, Mann-Whitney test, comparisons within genotypes. (**E**) Quantification of green fluorescent protein (GFP)–labeled cells in *Gr64f*;*CD8*-*GFP* flies crossed to *w1118^cs^* or *Gr64f*;*CD8-GFP > Pcl^RNAi^* on a control or sugar diet. *n* = 5 to 16, Kruskal-Wallis Dunn’s multiple comparisons, comparison to control diet of each genotype, no significance. (**F**) Triglyceride levels normalized to protein in *Gr5a > Pcl^RNAi^* and transgenic control flies fed a control or sugar diet. *n* = 8, two-way analysis of variance (ANOVA) with Sidak’s multiple comparisons test, comparisons to control diet within genotype. For all panels flies were on a control (circle) or sugar (square) diet for 7 days. All data are shown as means ± SEM, ****P < 0.0001 and *P < 0.05 for all panels.

We previously showed that restoration of sweet taste neuron activity in flies fed high dietary sugar protected them from diet-induced obesity (*14*, *15*), here defined by an increase in fat mass over protein levels. Since *Pcl* mutants abolished the deficits in neural and behavioral responses to sweetness in animals fed a high sugar diet, we anticipated that they should also prevent a diet-dependent increase in triglycerides. Sugar-diet flies with knockdown of *Pcl* in the *Gr5a^+^* neurons showed the same triglyceride levels as animals on a control diet (Fig. 2F), while these were increased in control flies fed a sugar diet (Fig. 2F). There was no difference in the levels of triglycerides between control and *Pcl* knockdown flies fed a control diet (Fig. 2F). Notably, mutants in the PRC2.1 complex were also protected from diet-induced obesity (fig. S2H). Together, these data suggest that, in response to the food environment, *Pcl* modulates the responsiveness of the sweet gustatory neurons to promote diet-induced obesity. The differences between PER and calcium responses to sucrose in *Pcl* mutant and control animals also suggest that the relative activity of the sensory neurons in response to diet rather than their absolute activity likely plays a role in taste sensation and diet-induced obesity.

### *Pcl* chromatin occupancy is redistributed in the high sugar environment

Our experiments show that PRC2.1 plays a critical role in the neural activity, behavior, and the metabolic state of animals exposed to the high sugar food environment. To identify the molecular mechanisms underlying these phenotypes, we measured the chromatin occupancy of *Pcl* in the ~60 *Gr5a^+^* neurons using targeted DNA adenine methyltransferase identification (Dam-ID) (TaDa) (Fig. 3A) (*23*). To do this, we generated *Dam::Pcl* (*UAS-LT3-Dam::Pcl*) transgenic flies and compared them to *Dam*-only flies (*UAS-LT3-Dam*) to control for nonspecific methylation by the freely diffusing *Dam* protein (Fig. 3A) (*24*) and to obtain a measure of chromatin accessibility in vivo Chromatin Accessibility profiling using Targeted DamID (CATaDa) (*25*). To specifically profile *Pcl* binding to chromatin in the sweet sensory neurons and limit the induction of *Dam*, we expressed the *Dam::Pcl* and *Dam* transgenes in combination with *Gr5a-GAL4*;*tubulin-GAL80^ts^*. To induce the expression of each UAS transgene, we shifted the flies to the permissive temperature (28°C) for 20 hours after they had been exposed to a control or sugar diet for 3 days (Fig. 3A). We selected this time point because we previously showed that sweet taste defects developed within 3 days of exposure to the sugar diet (*14*).

**Fig. 3:** *Pcl* chromatin occupancy is redistributed in the high sugar environment. (**A**) Targeted Dam-ID (TaDa) of *Dam::Pcl* and *Dam* (CATaDa) and induction paradigm. Age-matched *Gr5a*;*tubulin-GAL80^ts^ > UAS-LT3-Dam::Pcl* and *Gr5a*;*tubulin-GAL80^ts^ > UAS-LT3-Dam* flies were placed on a control or sugar diet for 3 days at 20° to 21°C and then switched to 28°C between days 3 and 4 to induce *Dam*. (**B**) CATaDa from control diet flies at the sweet gustatory receptor *Gr5a* and the bitter gustatory receptor *Gr66a*. (**C**) Proportion of genes allocated to the five chromatin states according to their transcription start site, normalized to the expected proportion across the whole genome. (**D**) Mean log_2_(*Dam::Pcl/Dam*) centered at PREs on a control and sugar diet. (**E** and **F**) Mean CATaDa centered at (E) *Pcl* peaks and (F) PREs on a control and sugar diet. (**G**) The median and variance of log_2_(*Dam::Pcl/Dam*) reads for genes differentially bound on a control and sugar diet. Genes are grouped into higher (group 1) or lower (group 2) *Pcl* binding on a sugar diet. (**H**) GO terms associated with differentially bound genes identified by iPAGE; boxes represent GO category, regulatory (lavender) and metabolism (orange) (for the full iPAGE, see fig. S4). For all panels, peaks are a false discovery rate (FDR) of <0.01.

Most of the variations in the biological replicates of *Dam::Pcl* normalized to *Dam* alone (see Materials and Methods) was due to diet (fig. S3A), consistent with high Pearson correlations within each dietary condition (fig. S3B). Further, the chromatin accessibility profile of *Dam* at the *Gr5a* sweet taste receptor gene promoter was high, while at the *Gr66a*, bitter taste receptor promoter—which is only expressed in bitter cells, closely located near the sweet cells—was low (Fig. 3B), suggesting that the transgenes were appropriately targeted to the sweet taste neurons and that the limited induction controlled for background DNA methylation.

We first analyzed *Pcl* chromatin occupancy in the *Gr5a^+^* neurons of flies on a control diet by comparing our data to a previous study that annotated five major chromatin types in *D. melanogaster* using a similar technique [DNA adenine methyltransferase identification (Dam-ID)] (*26*). *Pcl* targets were enriched in Polycomb chromatin (blue), compared to other chromatin types (green and black = repressive; red and yellow = active) (Fig. 3C); for example, *Pcl* occupancy was high and *Dam* accessibility low at two known Polycomb blue chromatin clusters (fig. S3E), while the opposite was true for regions in other chromatin types (fig. S3F, red and yellow chromatin) (*26*). We next asked whether *Pcl* was enriched at Polycomb response elements (PREs), cis-regulatory sequences to which Polycomb Group Proteins bind in *D. melanogaster* (*27*). Using a recently developed tool that predicts PRE regions genome-wide (*28*), we found that *Pcl* was present in these regions (Fig. 3D, gray line), with an enrichment for intergenic (3.2-fold enriched, *P* < 0.001, Monte Carlo permutation test) and enhancer PREs (2.9-fold enriched, *P* < 0.001, Monte Carlo permutation test).

To determine changes in *Pcl* occupancy with diet, we compared *Pcl* chromatin binding between flies fed a control and sugar diet. While ~70% of the overall *Pcl* and CATaDa peaks were shared between the control and sugar diet (fig. S3, C and D), we found more *Pcl* at PREs on a sugar compared to a control diet (Fig. 3D, purple line). Chromatin accessibility at both *Pcl* peaks (Fig. 3E) and PREs (Fig. 3F) was decreased under the sugar-diet condition. Our analysis also showed that differentially bound *Pcl* peaks had a 3.3-fold enrichment of overlap for enhancer-type PREs (*P* < 0.001, Monte Carlo permutation test). Further examination of the differentially bound genes revealed a redistribution in *Pcl* occupancy (fig. S3G), with a similar number of genes with higher (group 1) and lower (group 2) *Pcl* binding on a sugar diet compared to the control diet (Fig. 3G). To determine the biological pathways and function of the *Pcl* targets, we used iPAGE (*29*). This pathway enrichment analysis revealed that most of the genes differentially bound by *Pcl* were transcription factors that targeted promoters and enhancers. Notably, transcription factors implicated in axon target recognition and nervous system development showed an enrichment in *Pcl* binding on a sugar diet, while those involved in gene ontology (GO) terms such as proboscis development and feeding behavior had both an increase and decrease in *Pcl* occupancy on a sugar diet (Fig. 3H and, for full iPAGE GO term analysis, see fig. S4). While the large majority of genes differentially bound by *Pcl* was in the gene regulation category (80%), the pathway enrichment analysis also uncovered a few metabolism GO terms (Fig. 3H and fig. S4). In summary, we found that in the high sugar environment, the chromatin occupancy of PRC2.1 in the *Gr5a^+^* neurons was redistributed at loci that encode for transcription factors involved in neuronal processes and development. This redistribution could result in changes in the expression of these transcription factors and their targets and, in turn, affect the responsiveness of the sensory neurons and sweet taste sensation.

### PRC2.1 sculpts the transcriptional responses of the *Gr5a^+^* neurons in response to diet

To test the hypothesis that redistribution of PRC2.1 chromatin occupancy alters the physiology of the sensory neurons by changing gene expression, we used Translating mRNA Affinity Purification (TRAP) (*30*) to isolate mRNAs associated with the ribosomes of the *Gr5a*^+^ cells (Fig. 4A). To capture the dynamics of this process, we collected samples from age-matched *Gr5a > Rpl3-3XFLAG* flies fed a sugar diet for 3 and 7 days (fig. S5A). We first verified that this technique selected for mRNAs in the *Gr5a^+^* neurons by quantifying the normalized read counts (*Gr5a^+^*/input) for genes expressed only in the *Gr5a*^+^ cells, such as the sweet taste receptor genes *Gr5a*, *Gr64f*, and *Gr64a*, and the fatty acids taste receptor *Ir56D* (*31*, *32*). These transcripts were enriched in the *Gr5a^+^* fraction compared to the input (fig. S5B), while the opposite was true for the bitter receptor genes *Gr66a* and *Gr32a*, which are expressed in the bitter sensing neurons in the taste sensilla, but not in *Gr5a*^+^ cells (fig. S5B) (*33*).

**Fig. 4:** PRC2.1 sculpts the transcriptional responses of the *Gr5a^+^*neurons in response to diet. (**A**) TRAP to profile changes in the *Gr5a^+^* neurons. (**B** and **C**) Volcano plots representing differential expression in the *Gr5a^+^* neurons of age-matched male *Gr5a > UAS-Rpl3-3XFLAG* flies on SD3 (mint) and SD7 (teal). *n* = 2 to 3 replicates per condition. Nonsignificant genes are in black, and genes with *q* < 0.1 (Wald test) are in mint or teal for SD3 and SD7, respectively. (**D**) Venn diagram of DEGs at SD3, SD7, and the overlap between SD3 and SD7 (Wald test, *q* < 0.1). Heatmaps show l_2_fc for DEGs under each condition in Venn diagram (left columns in heatmap, SD3, SD7, and SD3 + SD7) and *Pcl^c429^* mutant flies (right column in heatmap, *Pcl^c429^* SD7). (**E**) Read counts from TRAP for *Pcl* bound (pink) and not bound genes (gray) on a control diet. Box plots represent median and variance, two-tailed *t* test, *P* = 3.196 × 10^−6^. (**F**) l_2_fc for *scarecrow* (*scro*), *Paired-like homeobox domain 1* (*Ptx1*), *caudal* (*cad*), *GATAe*, and *nubbin* (*nub*) in SD7, SD3 flies, and *Pcl^c429^*SD7 mutant flies. For all panels, comparisons are to control diet, and l_2_fc ranges from purple (high) to gold (low).

We observed a robust negative skew in gene expression in the *Gr5a*^+^ neurons of flies fed a sugar diet for 3 (SD3, mint; compared to the control diet) and 7 days (SD7, teal; compared to the control diet) (Fig. 4, B and C, −0.8 and −1.7 skew, refer to Materials and Methods for details of how skewness was calculated), consistent with the idea that a repressive gene regulatory mechanism is at play. Overall, we found ~800 differentially expressed genes (DEGs) at each time point (each compared to control diet, Wald test, *q* < 0.1; file S1), while ~190 were changed at both time points (Fig. 4D, Venn diagram, Wald test, *q* < 0.1); of these, ~68 and 87% showed negative log_2_ fold changes (l_2_fc), respectively (Fig. 4, B and C, SD3 and SD7). GO term analysis using iPAGE (*29*) revealed that these genes were part of biological pathways involved in three broad categories: neural function/signaling, metabolism, and gene regulation (figs. S6 and S7). GO terms for neuron-specific processes, such as dendritic membrane, sensory perception of chemical stimulus, and presynaptic/vesicle transport, were enriched at both time points (figs. S6 and S7), suggesting that PRC2.1 may alter the physiology of the sensory neurons through these pathways in response to a high sugar environment. Flies fed a sugar diet for 7 days showed additional changes in GO terms, specifically those linked to neurodevelopmental processes, such as asymmetric neuroblast division and neuron projection morphogenesis (fig. S7), which may explain the worsening of sweet taste sensation at this time point (*14*). We also observed changes in “regulatory” GO terms such as transcription factor and corepressor, consistent with the redistribution of *Pcl* chromatin occupancy in response to a high sugar diet that we had observed in the TaDa experiments (Fig. 3). Last, GO terms associated with metabolic changes were also higher in flies fed a sugar diet for 7 days (fig. S7), in line with the findings that longer consumption of the high sugar diet leads to higher fat accumulation (*14*). Together, this analysis shows that consumption of a high sugar diet altered neural, regulatory, and metabolic genes in the *Gr5a^+^* cells. Notably, the mRNA levels of the sweet taste receptors genes, *Gr5a*, *Gr64a-f*, and *Gr61a*, were not changed at either time point.

To determine the role of PRC2.1 in these changes, we performed the transcriptional profiling experiments in the *Gr5a^+^* neurons of *Pcl^c429^* mutant animals fed a control diet and sugar diet for 7 days (CD and SD7) (fig. S5C). Notably, the *Pcl* mutation abolished the negative skew (fig. S5D and file S1) and largely nullified the effects of the high sugar diet environment on differential gene expression. Specifically, of the genes repressed by a sugar diet (Fig. 4D, heatmap), 32% now had a positive l_2_fc (Wald test, *q* < 0.1), and 76% were unchanged (*q* < 0.1, practical equivalence test using a null hypothesis of a change of at least 1.5-fold; see Materials and Methods for details) between *Pcl* mutants fed a control and sugar diet. This effect was reflected in the GO analysis where terms changed by a high sugar diet in wild-type animals, such as dendritic membrane, sensory perception of chemical stimuli, synapse, and carbohydrate metabolic process, showed opposite trends in l_2_fcs in *Pcl* mutants (fig. S8). Thus, *Pcl* mutations abolished nearly all the gene expression changes induced by a high sugar diet consistent with their effects on behavior (PER; Fig. 1), neural function (in vivo calcium imaging; Fig. 2), and metabolism (triglycerides; Fig. 2). Together, these findings support the hypothesis that PRC2.1 tunes sweet taste sensation to the food environment by influencing the expression of genes involved in different aspects of sensory neuron physiology.

### PRC2.1 represses a transcriptional program required for sweet taste sensation

Our transcriptomics analysis shows that a high sugar diet environment repressed gene expression in the sweet sensory neurons and that *Pcl* mutations almost entirely abolished this effect. This, together with the finding that, on a sugar diet, *Pcl* binding primarily changed at the enhancers of transcription factor genes (Fig. 3), suggests that *Pcl* redistribution may affect the expression of transcription factors that control genes responsible for the overall responsiveness of these sensory neurons to sweetness. This idea is supported by the observation that *Pcl*-bound genes have lower expression levels than those not bound by it in the *Gr5a^+^* neurons (Fig. 4E), with many genes showing high binding and low expression [log_2_tpm+1 (Transcripts Per Kilobase Million) < 2; dark purple], while others having higher mRNA read counts (log_2_tpm+1 > 5; light purple) (fig. S5E). To test this hypothesis, we looked for transcription factors that were directly and differentially bound by *Pcl* in the TaDa analysis and that showed changes in gene expression on a sugar diet in the TRAP experiments (fig. S5F). This analysis revealed five genes: four transcriptional activators and one repressor. The four activators—*GATAe* (Zn finger), *nubbin/pdm* (*nub*; POU homeobox), *Ptx1* (paired-domain homeobox), and *caudal* (*cad*; hox-like homeobox)—had higher *Pcl* binding (Fig. 5A) and lower mRNA levels on a sugar diet (Fig. 4F). In contrast, the repressor *scarecrow* (*scro*; natural killer–like homeobox) had lower *Pcl* binding (Fig. 5A) and higher mRNAs levels on a sugar diet (Fig. 4F). Mutations in *Pcl* reversed the effects of a high sugar diet on the expression of these five genes, suggesting that the occupancy of *Pcl* to chromatin modulates their mRNA levels (Fig. 4F). We also noticed that *cad*, *Ptx1*, *GATAe*, and *nub* were enriched in the *Gr5a^+^* neurons compared to the input, while *scro* was depleted in these cells (fig. S9A). To test the effects of these five transcription factors on sweet taste, we manipulated their expression to mimic the direction of change on a high sugar diet. Knockdown of *cad*, *Ptx1*, *GATAe*, or *nub* and overexpression of *scro* (fig. S9, B and C) in the *Gr5a^+^* neurons of flies fed a control diet led to a decrease in sweet taste sensation (Fig. 5B) comparable to that experienced by animals on a sugar diet (Fig. 1B). These lines of evidence show that *cad*, *Ptx1*, *GATAe*, and *nub* are direct targets of PRC2.1 and necessary for sweet taste, while overexpression of *scro* is sufficient to decrease it. However, overexpression of each activator alone and knockdown of *scro* was not sufficient to rescue sweet taste in flies fed a sugar diet (fig. S9D).

**Fig. 5:** PRC2.1 represses a transcriptional program required for sweet taste. (**A**) Log_2_(*Dam::Pcl/Dam*) on a control and sugar diet within a 50-kb window at *cad*, *Ptx1*, *GATAe*, *nub*, and *scro*. Replicates are superimposed. Turquoise traces are SD/CD fold changes. Peaks are black boxes (*q* < 0.01), genes are in dense format to include all isoforms. (**B**) Taste responses (*y* axis) to stimulation of the labellum with 30, 10, and 5% sucrose (*x* axis) in age-matched males of *Gr5a > cad^RNAi^*, *Gr5a > Ptx1^RNAi^*, *Gr5a > GATAe^RNAi^*, *Gr5a > nub^RNAi^*, *Gr5a > scro*, and transgenic control flies on a control diet for 7 days. *n* = 30 to 54. All data shown as means ± SEM. ***P < 0.0001, ***P < 0.001, and **P < 0.01. (**C**) l_2_fcs for candidate gene targets of Cad, Ptx1, GATAe, Nub, and Scro (see Materials and Methods and fig. S9) at SD7 and *Pcl^c429^* mutants at SD7. (**D**) Venn diagram of the overlap of the candidate gene targets of Cad, Ptx1, GATAe, Nub, and Scro. (**E**) Transcriptional loop between Cad, Ptx1, GATAe, Nub, and Scro mediated by Pcl. GO terms associated with each regulator and identified by iPAGE. Boxes represent GO category, metabolism (orange), regulatory (lavender), and neural/signal (blue) (for full iPAGE see file S1). cAMP, cyclic adenosine 3′,5′-monophosphate.

Given that the four activators are required for sweet taste sensation, we reasoned that they may control the expression of genes important for the proper function of the *Gr5a^+^* neurons and normal sweet taste. To identify candidate target genes, we tiled the entire *D. melanogaster* genome using the motifs for each of these five transcription factors, converted the hits for each transcription factor to *z* scores, and determined candidate regulatory targets based on estimates of the *z* score threshold for binding in each case (see Materials and Methods for details and fig. S9E). We then flagged as “targets” the set of genes that had a putative binding site (exceeding our transcription factors-specific *z* score cutoff) within a 2-kb region upstream of the annotated open reading frame start (fig. S9, F to J) and examined their expression pattern in the *Gr5a^+^* neurons of flies on a control and sugar diet. This analysis revealed 658 genes that were collectively regulated by these five transcription factors and also changed by a high sugar diet in the *Gr5a^+^* cells (file S1). Targets of the transcriptional activators Cad, Ptx1, GATAe, and Nub—which *Pcl* repressed on a sugar diet—showed negative l_2_fcs on a sugar diet (Fig. 5C, SD7, teal) that reverted in the *Pcl* mutants (Fig. 5C, pink). Conversely, targets of the transcriptional repressor Scro—which was released from *Pcl* binding and had higher mRNA levels on a sugar diet—showed negative l_2_fcs on a sugar diet (Fig. 5C, SD7, teal) that reverted in *Pcl* mutants (Fig. 5C, pink). Notably, the 658 putative targets of these five transcription factors accounted for nearly all the genes changed by a high sugar diet as measured by TRAP (Fig. 4), suggesting that by directly modulating the expression of these five regulators—*Ptx1*, *cad*, *GATAe*, *nub*, and *scro—*and their targets, PRC2.1 influences the responsiveness of the *Gr5a^+^* neurons to sweet stimuli and the animal’s taste sensation.

We next asked whether these targets may be cooperatively regulated by these five transcription factors. We observed a significant overlap among the regulons of all of the four transcriptional activators [Fisher’s exact test, false discovery rate (FDR)–corrected *P* < 0.000001] with the exception of *Ptx1-Nub* (Fig. 5D), suggesting that, together, the four transcription factors suppressed by PRC2.1 in the high sugar diet environment, may cooperate to direct the expression of a common set of target genes. We also found evidence for *Scro* binding sites in the genes targeted by the four activators (Fisher’s exact test, *q* < 0.000001). This is interesting because binding of Scro to these gene targets could ensure a more direct and robust way to repress them compared to PRC2.1 only silencing their activators (*cad*, *Ptx1*, *GATAe*, and *nub*). Notably, this double repression mechanism, the first via Pcl and the second via Scro, could explain the large negative skew in gene expression on a high sugar diet we observed in the TRAP data. Transcription factors that share common targets are often part of feed-forward loops, where they regulate one another and themselves to ensure stability of gene expression. We found that *GATAe* had predicted binding sites in the promoters of all four regulators considered here (*cad*, *scro*, *Ptx1*, and *nub*), in addition to binding its own promoter in an autoregulatory loop (Fig. 5E). Furthermore, our predictions show that Cad also could target itself, Ptx1 could target *nub*, and that Scro may regulate both *cad* and *GATAe*, forming a negative feedback loop with the latter (Fig. 5E and file S1). Thus, the five transcription factors differentially bound by PRC2.1 between diets form a hub that regulates the physiology of the *Gr5a^+^* neurons.

To probe which aspects of physiology were changed, we used pathway enrichment analysis on the regulons for each transcription factor. GATAe targets, which comprise a large number of the genes targeted by the four other transcription factors, were enriched for GO terms involved in synaptic assembly and growth, terminal bouton, neural projection morphogenesis, and protein kinase regulation (summarized in Fig. 5E and file S1). In contrast, Ptx1 targets were enriched in GO terms implicated in cyclic adenosine 5′-monophosphate signaling, detection of chemical stimuli, and morphogenesis (summarized in Fig. 5E and file S1), Cad targets showed enrichments in GO terms adenylate cyclase activity, sensory perception, and neuropeptide signaling (summarized in Fig. 5E and file S1), and Nub targets in calcium signaling and nucleosome (summarized in Fig. 5E and file S1). The targets of the repressor Scro showed enrichment in both neural and metabolic GO terms such as olfactory behavior and carbohydrate metabolic process (summarized in Fig. 5E and file S1). Thus, we predict that these transcriptional regulators may contribute to different aspects of the physiology of the *Gr5a*^+^ cells.

To test the possibility that these targets form a functional network, we used STRING, a database of known and predicted physical and functional protein-protein interactions (*34*). We found a significant number of interactions above the expected number (protein-protein interaction enrichment of *P* < 1.0 × 10^−16^; file S2), suggesting that the targets may be part of a functional and biologically connected network in the *Gr5a^+^* neurons. We used a subset of the targets with GO terms in neural processes to build a smaller network to identify target genes that may play a direct role in sweet taste sensation. This network showed strong interactions between genes involved in synaptic organization and signal transduction and their connection with the upstream regulators (fig. S10A). We chose two genes at the edges of the network, which are less likely to have redundant functions, the *adenylyl cyclase X D* (*ACXD*) gene (*35*) and the *activity-regulated cytoskeleton–associated protein 1* (*Arc1*) (*36*). Knockdown and mutations of *Arc1* or *ACXD* in the *Gr5a^+^* cells of flies on a control diet led to a significant decrease in sweet taste responses compared to the transgenic controls (fig. S10, B to D). Together, these lines of evidence suggest that PRC2.1 mediates the effects of a high sugar diet on sweet taste by directly controlling the expression of a developmental transcriptional program important for sweet taste.

### The persistent phenotypic memory of the food environment is dependent on PRC2.1

The cellular fates created by Polycomb Group Proteins are inherited as memories across cell divisions to ensure phenotypic stability even in the absence of the triggering stimuli (*37*, *38*). We therefore asked whether the neural and behavioral state created by PRC2.1 in the high sugar diet environment was maintained when flies were moved to the control diet for different durations (7, 10, 15, and 20 days) after eating a sugar diet for 7 days (SD > CD) (Fig. 6, A and B, and fig. S10E). We found that animals switched to the control diet still had a dulled sweet taste, similar to that of age-matched flies fed a sugar diet for 7 days (Fig. 6B, top, SD7 > CD7 compared to CD7 > SD7). However, their triglyceride levels were similar to those of control diet flies (fig. S10F), suggesting that while fat storage was reversible when flies were switched to the “healthy” control diet, sweet taste deficits persisted for up to 20 days (fig. S10E). We also found that persistence required exposure to the high sugar diet for at least 5 days (fig. S10G).

**Fig. 6:** The persistent phenotypic memory of the food environment is dependent on PRC2.1. (**A**) Control (CD7), sugar (SD7) diet, and > represents dietary switch. (**B**) Taste responses (*y* axis) to stimulation of the labellum with 30, 10, and 5% sucrose (*x* axis) of age-matched male *w1118^cs^* flies on a control (CD7 > CD7), control to sugar (CD7 > SD7), and sugar to control (SD7 > CD7) diet without (top; *n* = 57 to 64) or with EEDi (bottom; *n* = 34 to 46); comparisons between CD7 > CD7 and CD7 > CD7 + EEDi (ns), CD7 > SD7 and CD7 > SD7 + EEDi (****), and SD7 > CD7 and SD7 > CD7 + EEDi (****). Data are shown as means ± SEM. ****P < 0.0001 and **P < 0.01. (**C**) Differential expression in the *Gr5a^+^* neurons of age-matched male *Gr5a > UAS-Rpl3-3XFLAG* flies on a sugar to control (SD7 > CD7) diet compared to control diet (CD7 > CD7), *q* < 0.1 (green), ns is nonsignificant, *n* = 2 replicates per condition. (**D**) Heatmap of DEGs from Fig. 5C that change in the SD7 > CD7 (48%) compared to SD3, SD7, and *Pcl^c429^*SD7. l_2_fc ranges from purple (high) to gold (low). (**E**) Model of molecular changes in the *Gr5a^+^* neurons on a control and sugar diet, showing the redistribution of PRC2.1, and the effects on the regulators and neural responses to sweetness.

To understand how this phenotype compares to that of the control diet flies at the molecular level, we conducted TRAP of the *Gr5a^+^* neurons of flies under the SD7 > CD7 and CD7 > CD7 conditions. mRNAs from flies on a SD7 > CD7 showed a strong negative skew in overall l_2_fc compared to the control diet group (Fig. 6C, −2.06), reminiscent of the skew we observed in flies fed a sugar diet (Fig. 4C). Furthermore, we observed that 47% (310 of 658) of genes in the transcriptional network repressed by PRC2.1 on a sugar diet were still decreased in SD7 > CD7 flies (Fig. 6D). The SD7 > CD7 animals clustered with the SD7 group compared to SD3 and *Pcl* mutants fed a sugar diet (Fig. 6D). Thus, half of the transcriptional state established by dietary sugar via PRC2.1 persisted even after the dietary environment was changed.

To test the hypothesis that PRC2.1 plays an active role in the maintenance of this transcriptional state in the absence of the sugar diet, we inhibited PRC2 activity during the “recovery” diet using the EEDi inhibitor (SD7 > CD7 + EEDi). These animals showed a restoration of wild-type sweet taste (Fig. 6B, bottom, green diamonds) compared to flies fed the recovery diet without EEDi supplementation (Fig. 6B, top, SD7 > CD7). Similarly, knockdown of *Pcl* only during the recovery period using the temperature sensing *tubulin-GAL80^ts^*, also rescued sweet taste deficits (fig. S10H). Together, these data indicate that the sensory neurons retain a transcriptional and phenotypic memory of the sugar diet environment that leads to long lasting behavioral deficits. Further, our findings suggest that the persistence of this state is actively maintained and requires the constitutive action of PRC2.1.

## DISCUSSION

In this study, we set out to understand how dietary experience alters the gustatory system to promote food intake and weight gain. Specifically, we took advantage of the simple sensory system of *D. melanogaster* and its exquisite genetic and neural tools to identify the molecular mechanisms through which diet composition changes neural physiology and behavior. We previously found that high dietary sugar decreased the responsiveness of the sensory neurons to sugar stimuli, leading to a dulled sense of sweet taste, independently of fat accumulation (*14*). Here, we show that the decrease in sweet taste sensation that flies experience after chronic exposure to a high sugar diet is caused by the cell-autonomous action of the PRC2.1 in the sweet gustatory neurons. Mutations and pharmacological inhibition of PRC2.1 blocked the effects of the food environment on neural activity, behavior, and obesity. While we do not exclude the possibility that PRC1 and Pho RC may also be involved, we found that mutations or knockdown in these complexes had no effect on taste. To this point, we observed that in *Pcl* mutants, even if the neural responses to sucrose were identical in control and sugar diet fed flies, they were of lower in magnitude than those of control flies, suggesting that PRC2.1 may modulate plasticity bidirectionally in response to the nutrient environment. This also suggests that, within limits, it is the relative rather than the absolute output of the sensory neurons that is important for taste sensation and diet-induced obesity.

In the high sugar food environment, PRC2.1 chromatin occupancy was redistributed, leading to the repression of transcription factors, neural, signaling, and metabolic genes that decreased the responsiveness of the *Gr5a^+^* neurons and the fly’s sensory experience of sweetness. However, we found that PRC2.1 did not directly bind to neuronal genes in these cells and that, instead, it targeted transcription factors involved in processes such as sensory neuron development, synaptic function, and axon targeting. Specifically, on a high sugar diet *Pcl* binding was increased at the loci of transcription factors *cad*, *GATAe*, *nub/pdm*, *Ptx1* and decreased at the *scro* locus and lead to corresponding changes in the mRNA levels of these genes (Fig. 6E, model). Our computational analysis revealed that in the *Gr5a^+^* cells, these five transcriptional factors regulate a network of ~658 candidate target genes implicated in synaptic function, signal transduction, and metabolism. Changes in the levels of the five transcription factors on a high sugar diet resulted in the overall repression of their target genes, providing a possible explanation for the alterations in the responsiveness of the *Gr5a^+^* cells we observed. We predicted several positive and negative regulatory loops among the five transcription factors, suggesting that they could form a regulatory hub that is responsive to changes in the dietary environment. Knockdown of the four activators and a few of their targets and overexpression of *scro* (Fig. 5B and fig. S10, B to D) resulted in a decrease in sweet taste on a control diet. However, overexpression of *cad*, *Ptx1*, and *nub* alone and knockdown of *scro* did not rescue taste deficiencies in animals fed a high sugar diet. Since there is (i) overlap in the predicted targets between the repressor *scro* and each of the four activators and (ii) overlap among the targets of the four activators, one possibility is that, as long as *scro* levels are higher because of the sugar diet, the repressive drive is so strong that overcoming it requires collaborative binding among the activators. Together, these findings suggest that this transcriptional hub and the gene battery it controls are necessary for sweet taste and reshaped by high dietary sugar.

How do these transcription factors and their targets modulate the physiology of these gustatory neurons? Several of these transcription factors (*Ptx1*, *scro*, and *nub/pdm*) control the proper branching, synaptic connectivity, and activity of sensory neurons (*39*–*45*), while others (*cad* and *nub/pdm*) play a role in neuroblast development (*46*); PRC2 also functions as a competence factor in neural proliferation, differentiation, and sensory neurons (*39*, *46*, *47*). We propose that the gene battery of ~658 genes controlled by this transcriptional hub may define the intrinsic properties of the sweet sensing neurons. We observed that the four activators that are repressed by *Pcl* under the high sugar condition are enriched in the *Gr5a^+^* cells while *scro* is depleted. Further, many of the target genes are involved in signaling, synaptic function, and cell adhesion, including the kinase *haspin*, the adenylate cyclase *ACXD*, *syt-alpha*, *Arc1*, and the tetraspanin, jonan, and innexin proteins among others. These genes were part of a highly interconnected network, which could affect the responsiveness and wiring of the sweet gustatory neurons. Since we did not observe a change in the expression of the sweet taste receptors, or the misexpression of other taste receptors, our data are not consistent with a complete “loss” of identity of the *Gr5a^+^* neurons with a high sugar diet. Instead, we hypothesize that PRC2.1 tunes these sensory neurons to the dietary environment by altering a developmental transcriptional network that controls the intrinsic properties of the *Gr5a^+^* cells, particularly those involved in signal transduction, connectivity, synaptic function, and metabolism. Studies that test the effects of this network on the wiring, morphology, and signal transduction of the sweet sensory neurons will shed light on how exactly the transcriptional remodeling caused by PRC2.1 we found here affects the physiology of *Gr5a^+^* cells.

While our experiments show that PRC2.1 chromatin occupancy shifts with the dietary environment, we did not define the signaling mechanisms through which this change in binding occurs. Thus, the question of how exactly PRC2.1 binding is altered in response to the food environment remains open. Recent studies suggest that the activity of Polycomb Group Proteins is directly and indirectly linked to cellular metabolism, including kinase signaling cascades, the post-translational modification O-linked-N-acetylglucosaminylation (GlcNAcylation), and the availability of cofactors for histone modifications (*48*, *49*). Our previous work showed that the hexosamine biosynthesis pathway enzyme *O-GlcNAc transferase* (*OGT*) acts in the *Gr5a^+^* neurons to mediate the effects of high dietary sugar on sweet taste (*14*); whether the interaction between OGT and PRC2 is what promotes the repressive activity of the latter in these sensory neurons is a question worth investigating. Notably, the dysregulation of Polycomb-associated chromatin has been found in mice and humans with diet-induced obesity (*50*, *51*), suggesting that the mechanisms we found here could also underlie the chemosensory alterations reported in mammals (*8*–*12*).

More broadly, our work opens up the exciting possibility that PRC2 may modulate neural plasticity in response to environmental conditions by reengaging developmental programs. Despite its central role in development and maintenance of neural identity, studies have not directly linked PRC2.1 to neural plasticity. However, in other postmitotic cells such as muscle, Polycomb Group Proteins are known to reshape transcriptional programs according to environmental stressors, such as oxidative stress, injury, temperature, and light (*48*, *49*). Our findings advance the conceptual understanding of the role of Polycomb Group Proteins in the nervous system and suggest that they could also modulate “neural states” and metaplasticity in response to environment stimuli. Using neuroepigenetic mechanisms such as those used by Polycomb Group Proteins to tune neural states to external conditions could provide several advantages compared to the medley of other cellular-, receptor-, or synaptic plasticity–based mechanisms. Specifically, it would allow cells to (i) orchestrate a coordinated response, (ii) create a memory of the environment, and (iii) buffer small fluctuations until a substantial challenge is perceived. It is particularly fascinating to think about the molecular mechanisms through which these neural states may be established. The need of neurons to constantly maintain their identity may mean that environmental signals such as the extent of sensory stimulation could alter the expression of developmental gene batteries and affect neural physiology (*52*). It has been speculated that some forms of plasticity may reengage developmental programs that specify the intrinsic properties of neurons (*53*). Here, we observed that the regulators of the transcriptional network we uncovered function in sensory neuron development and are enriched in the *Gr5a^+^* cells. Thus, it could be a hallmark of neuroepigenetic plasticity to exploit developmental programs, linking the known role of PRC2 in establishing cell fates with this newly found function in modulating cell states.

Incidentally, reengaging developmental programs could be the reason why some environments and experiences leave a memory that leads to the persistent expression of the phenotype beyond the presence of the triggering stimulus, as these could target neural connectivity and set synaptic weight thresholds. Here, we found that the changes in taste sensation and half of the sugar diet neural state set by PRC2.1 remained even after animals were moved back to the control diet for up to 20 days. A limitation of our study is that because of the small number of *Gr5a^+^* neurons and their anatomically inaccessible location, we were not able to measure the identity of the molecular memory in these cells alone. However, we saw that the phenotypic memory of the high sugar food environment was dependent on the constitutive action of PRC2.1. Thus, on the basis of other studies showing that the H3K27 methyl mark acts as a molecular memory during development (*37*, *38*), we speculate that this is likely to be the memory signal in the *Gr5a^+^* cells too. Stable maintenance of the memory requires active recruitment of PRC2 (*38*); while we did not measure *Pcl* occupancy and chromatin accessibility at PREs in the recovery diet with and without the inhibitor, our findings that PRC1.2 is actively required for the maintenance of the taste phenotype and that 47% of its indirect targets are still repressed indicate that PRC2.1 may be stably recruited to the transcription factors loci. Perhaps, conditions that lead to metabolic remodeling such as prolonged fasting could reset its binding. Last, we do not know whether the diet-induced chemosensory plasticity observed in humans and rodents is persistent or reversible. Unlike in *D. melanogaster*, mammalian taste cells are not postmitotic neurons, and so, they regenerate every few weeks. Thus, the persistence of chemosensory plasticity in mammals, if it exists, may involve different mechanisms in the taste cells, such as a decrease in their renewal (*8*) or changes in their wiring to sensory neurons. However, a decrease in the responses of the chorda tympani to sweetness has been observed in rats fed a 30% sucrose diet (*12*), and thus, our findings may be applicable to the sensory nerves.

In conclusion, we show that PRC2.1 mediates the effects of high dietary sugar on sweet taste by establishing persistent alterations in the taste neurons that remain as a phenotypic and transcriptional memory of the previous food environment. We speculate that this memory may lock animals into patterns of feeding behavior that become maladaptive and promote obesity. Thus, dietary experience, in ways like trauma, can induce lasting molecular alterations that restrict the behavioral plasticity of animals and affect disease risk. Since the content of sugar in processed foods is similar to that we fed flies and the function of Polycomb Group Proteins is conserved from plants to humans (*16*), our work is broadly relevant to understanding the effects of processed food on the mammalian taste system and its impact on food intake and a whole range of diet-related conditions and diseases that affect billions of people around the globe.

## MATERIALS AND METHODS

### Fly husbandry and strains

All flies were grown and maintained on cornmeal food (Bloomington Food B recipe) at 25°C and 45 to 55% humidity under a 12-hour light/12-hour dark cycle (Zeitgeber time 0 at 9:00 a.m.). Male flies were collected under CO_2_ anesthesia 1 to 3 days after eclosion and maintained in a vial that housed 35 to 40 flies. Flies were acclimated to their new vial environment for an additional 2 days. For all experiments, flies were changed to fresh food vials every other day.

For all dietary manipulations, the following compounds were mixed into standard cornmeal food (Bloomington Food B recipe) (0.58 calories/g) by melting, mixing, and pouring new vials as in (*54*) and (*55*). For the 30% sugar diet (1.41 calories/g), Domino granulated sugar (w/v) was added. For the EEDi inhibitor diet (Axon Medchem), EEDi was solubilized in 10% DMSO and added to control or 30% sugar diet at a total concentration of 5 or 8 μM.

For genetic manipulations, the GAL4/UAS system was used to express transgenes of interest in *gustatory receptor 5a Gr5a-GAL4*. For each GAL4/UAS cross, transgenic controls were made by crossing the *w1118^CS^* (gift from A. Simon, *CS* and *w1118* lines from the Benzer laboratory) to GAL4 or UAS flies, sex-matched to those used in the GAL4/UAS cross. PRC2 mutants were not in a *w1118^CS^* background but were crossed to this line for all experiments shown here. The fly lines used for this paper are listed in file S1.

### Proboscis extension response

Male flies were food deprived for 18 to 24 hours in a vial with a Kimwipe dampened with 2 ml of Milli-Q filtered deionized water. PER was carried out as described in (*17*). Extension responses were scored manually, and when possible, blind observers were used.

### Proboscis immunofluorescence

Probosces were dissected in 1× phosphate-buffered saline and fixed in 4% paraformaldehyde, mounted in FocusClear (CelExplorer) on coverslips. Cell bodies were imaged using an FV1200 Olympus confocal with a 20× objective. Cells were counted using Imaris Image Analysis software.

### Triglyceride measurements

Total triglycerides normalized to total protein were measured as described in (*14*). Briefly, two flies per biological replicate were homogenized in lysis buffer [140 mM NaCl, 50 mM tris-HCl (pH 7.4), and 0.1% Triton X-100] containing protease inhibitor cocktail (Thermo Fisher Scientific). Lysate extract was used to determine protein and triglyceride concentrations using Pierce bicinchoninic acid (BCA) assay (Thermo Fisher Scientific; absorbance, 562 nm) and Triglycerides LiquiColor Test (Stanbio; abs, 500 nm), respectively.

### Calcium imaging

Male flies expressing *GCaMP6s-Brp-mCherry* (*22*) in the sweet-sensing neurons were food deprived for 18 to 24 hours. Flies were imaged as in (*14*): briefly, animals were fixed to a custom-printed plastic slide with paraffin wax, and the proboscis was waxed to an extended position. Distal leg segments were removed to prevent tarsal interference with labellar stimulation. To image the subesophageal zone (SEZ), a sugarless artificial hemolymph solution filled the well surrounding the head. Subsequently, the dorsal cuticle between the eyes was removed by microdissection to expose the brain. Each fly proboscis was tested with Milli-Q water before stimulating with 30% sucrose dissolved in Milli-Q water. Stimulus (a piece of Kimwipe soaked in tastant and held with forceps) delivery to the proboscis was manual and timed to coincide with the 100th recording sample of each time series. Imaging was carried out using an upright confocal microscope (Olympus, FluoView 1200 BX61WI), a 20× water-immersion objective and a laser excitation at 488 and 543 nm. Recordings were made at 4 Hz (512 × 512 pixels). Plane of interest was kept to the most ventral neuropil regions innervated by the sweet-sensing neurons. Percent Δ*F*/*F*_0_ was calculated for regions of interest (ROIs) enclosing the *Gr64f^+^* neuropil regions in the SEZ, one per hemisphere. To calculate %Δ*F*/*F*_0_, the ROI intensity during the 10 frames preceding stimulus delivery was averaged to create the baseline intensity value *F*_0_. The baseline value was then subtracted from the ROI intensity value in each frame (*F*), and the result (Δ*F*) was then divided by the baseline and multiplied by 100 to produce %Δ*F*/*F*_0_. The red channel %Δ*F*/*F*_0_ was subtracted from the green channel %Δ*F*/*F*_0_ for each frame to correct for movement. For all flies, there were no detectable taste responses in the red channel. Peak %Δ*F*/*F*_0_ for each fly was determined by selecting the highest %Δ*F*/*F* response after stimulation.

### RNA extraction and quantitative reverse transcription polymerase chain reaction

For all RNA extractions used for quantitative polymerase chain reaction (qPCR), heads from 10 to 20 flies were dissected into TRIzol (Ambion) and homogenized with plastic pestles. RNA was extracted by phenol chloroform (Ambion) and precipitated by isopropanol with GlycoBlue Coprecipitant (Invitrogen). RNA pellet was washed as needed with 75% ethanol, subsequently eluted in nuclease-free water, and treated with deoxyribonuclease I (DNase I), according to the manufacturer’s instructions (Turbo DNA-free DNA Removal Kit, Ambion). All steps were carried out under ribonuclease (RNase)–free conditions, and RNA was stored at −80C until further processing.

Complementary DNA was synthesized by SuperScript III (Invitrogen) reverse transcriptase with the addition of RiboLock RNase Inhibitor (Thermo Fisher Scientific). qPCR reactions were carried out using Power SYBR Green PCR Master Mix (Applied Biosystems) based on the manufacturer’s instructions. Primers were added at a 2.5 μM concentration. All reactions were run on a 96-well plate on the StepOnePLus Real-Time PCR System (Applied Biosystems), and quantifications were made relative to the reference gene ribosomal protein 49 (Rp49). Primer sequences are listed in file S1 and were tested for efficiency before the qPCR experiment. Relative fold changes in transcript abundance were determined with the Livak method using the Rp49 transcript as a housekeeping control.

### Affinity purification of ribosome-associated mRNA

Three hundred heads (10,000 *Gr5a^+^* cells) per biological replicate were collected using prechilled sieves in liquid nitrogen on dry ice. Frozen heads were then lysed as previously described (*30*). From the lysate, total RNA was extracted by TRIzol LS Reagent (Thermo Fisher Scientific, 10296010) as input. The remainder of the lysate was incubated with Dynabeads Protein G (Thermo Fisher Scientific, 10004D) to preclear samples for 2 hours and subsequently incubated with Dynabeads Protein G coated with an anti-Flag antibody (Sigma-Aldrich, F1804). The lysate-beads mixture was incubated at 4°C with rotation for 2 hours, then. RNA was extracted from ribosomes bound to the beads by TRIzol Reagent (*30*).

### Targeted DNA adenine methyltransferase identification

For the *UAS-LT3-Dam::Pcl* construct, the coding region of the *Pcl* gene was amplified from the *pCRE-NDAM-Myc-DO69-Pcl* (gift from B. Van Steensel, The Netherlands Cancer Institute) with primers listed in file S1 and assembled into the *UAS-LT3-DAM* plasmid (gift from A. Brand, University of Cambridge) using the NEBuilder HiFi DNA Assembly kit based on the manufacturer’s instructions [New England Biolabs (NEB)]. Transgenic animals were validated by reverse transcription PCR for correct insert. These lines were crossed to *Gr5a-GAL4*;*tubulin-GAL80^ts^*. All animals were raised and maintained at 20°C. Expression of *Dam::Pcl* and *Dam* was induced at 28°C for 18 to 20 hours. For all experiments, 300 heads of males and female flies were collected per replicate on dry ice by sieving. DNA was extracted from frozen heads following kit instructions (Invitrogen). For identification of methylated regions, purified DNA was digested by Dpn I, followed by PCR purification of digested sequences. TaDa adaptors were ligated by T4 DNA ligase (NEB). Adapter ligated DNA was PCR-amplified according to the protocol (*23*) and subsequently purified. Purified DNA was digested with Dpn II, followed by sonication to yield fragments averaging 200 to 300 base pairs (bp). TaDa adaptors were removed from sonicated DNA by digestion. Sonicated DNA is used for library preparation (*23*).

### Library preparation for TRAP and TaDa

RNA sequencing (RNA-seq) libraries were generated using the Ovation SoLo RNA-seq System for *Drosophila* (NUGEN, 0502-96). All reactions included integrated Heat-Labile Double-Strand Specific DNase treatment (ArcticZymes, catalog no. 70800-201). DNA-sequencing libraries were generated using the Takara ThruPLEX Kit (catalog no. 022818) using 3-ng input and 10 cycles of PCR. All libraries were sequenced on the Illumina NextSeq platform (High-output kit v2 75 cycles) at the University of Michigan core facility.

### High-throughput RNA-seq analysis

Fastq files were assessed for quality using FastQC (*56*). Reads with a quality score below 30 were discarded. Sequencing reads were aligned by STAR (*57*) to dmel-all-chromosomes of the dm6 genome downloaded from Ensemble, and gene counts were obtained by HTSeq (*58*). Count files were used as input to call differential RNA abundance by DESeq2 (*59*). To determine the efficiency of the TRAP, experiment pairwise comparisons were made between the *Gr5a^+^*-specific IP fraction and the input, where the numerator is the immunoprecipitation (IP) and the denominator is the input. For comparisons between dietary conditions, DESeq2 was only applied to the *Gr5a^+^*-specific IP condition. All pairwise comparisons were made to the control diet of the corresponding genotypes, such that SD3 and SD7 were compared to the age-matched control diet group. In *Pcl* mutant experiments, the pairwise comparison was made between sugar diet and control diet within the age-matched *Pcl^c429^* genotype group. A cutoff of *q* < 0.1 was used to call DEGs. Skew in l_2_fcs was measured using the R package Skewness (e1071). The skew determination is based on the l_2_fc for all genes detected, not only those that are significantly differentially expressed. Skewness is calculated from the second and third central moments of the observed distribution of l_2_fcs, using the formula Skewness = *m*_3_/(*m*_2_)^3/2^, where *m*_r_*r* = Σ*_i_* (*x_i_* − μ)*^r^*/*n*, with *r* as an integer, *i* indexing the data observations (genes in this case), and *n* as the total number of observations (genes). In general, a negative skewness in a unimodal distribution indicates the presence of a long negative tail, whereas a positive skewness indicates a long positive tail. RNA-seq data visualization was carried out in R studio using ggplot2 and the following packages, pheatmap (*60*), Venneuler (*61*), and EnhancedVolcano (*62*). To cluster columns and rows in pheatmap, “Ward.D” clustering was used.

### High-throughput TaDa and CATaDa analysis

Fastq files were assessed for quality using FastQC (*56*). Reads with a quality score below 30 were discarded. The damidseq_pipeline was used to align, extend, and generate log_2_ ratio files (*Dam::Pcl/Dam*) in GATC resolution as described previously (*63*). Briefly, the pipeline uses Bowtie2 (*64*) to align reads to dmel-all-chromosomes of the dm6 genome downloaded from Ensemble, followed by read extension to 300 bp (or to the closest GATC, whichever is first). Bam output is used to generate the ratio file in bedgraph format. Bedgraph files were converted to bigwig and visualized in the UCSC Genome Browser. Correlation coefficients and principal components analysis plot between biological replicates were computed by multibigwigSummary and plotCorrelation in deepTools (*65*). Fold-change traces for SD/CD of log_2_(*Dam::Pcl/Dam*) were generated by calculating the mean coverage profile of all replicates for each condition and subsequently calculating fold change between the sugar diet and control diet condition with deepTools bigwigCompare (*65*). Peaks were identified from log_2_(*Dam::Pcl/Dam*) ratio files using find_peaks (FDR, <0.01) (*63*). To do this, the binding intensity thresholds are identified from the dataset, the dataset is then shuffled randomly, and the frequency of consecutive regions (i.e., GATC fragments or bins) with a score greater than the threshold is calculated. The FDR is the observed/expected for a number of consecutive fragments above a given threshold. Association of genes to peaks was made using the peaks2genes script (*63*) and dm6 genome annotations. Overlapping intervals or nearby intervals were merged into a single interval using mergeBed in BEDtools (*66*). Intervals common in all replicate peak files were identified by Multiple Intersect in BEDtools (*66*). DiffBind was used to determine differentially bound sites on peak files based on differences in read intensities (*67*). All differentially bound sites are shown in file S1. For CATaDa experiments, all analyses were performed similar to those of TaDa with the exceptions that (i) *Dam* only profiles were not normalized as ratios but shown as non-normalized binding profiles, (ii) *Dam* only coverage plots were generated by converting bam files to bigwig files normalized to 1× dm6 genome, and (iii) peaks were called using MACS2 (Model-based Analysis of ChIP-Seq 2) call peaks on alignment files without building the shifting model with an FDR of <0.05 (*68*). To determine the proportion of genes that fit within the various chromatin domain subtypes, we first matched *Dam::Pcl/Dam* targets to coordinates identified by (*26*) and then determined their gene count in each chromatin subtype (observed) compared to the whole genome (expected). All chromatin tracks are visualized with the UCSD genome browser.

### iPAGE analysis

All GO term enrichment analysis was performed using the iPAGE package (*29*), using gene-GO term associations extracted from the Flybase dmel 6.08 2015_05 release (*69*). For analysis of TRAP data, iPAGE was run in continuous mode, with l_2_fcs divided into seven equally populated bins. For analysis of TaDa and Transcription Factor (TF) regulatory targets, iPAGE was run in discrete mode, using the groups specified for each calculation. For all discrete calculations, independence filtering was deactivated because of the less informative available signal. All other iPAGE settings were left at their default values. All shown GO terms pass the significance tests for overall information described in (*29*); in addition, for each term, individual bins showing especially strong contributions [*P* value, such that a Benjamini-Hochberg FDR (*70*) calculated across that row, yields *q* < 0.05] are highlighted with a strong black box.

### PREdictor

Identification of predicted PRE sites was performed exactly as described in (*28*), using the dm6 genome. As suggested in (*28*), we use a threshold confidence score of 0.8 to identify the PREs used in the present analysis. A complete list of predicted PREs, with accompanying confidence scores, is shown in file S1. Enrichments of overlap between different PRE classes and *Pcl* occupancy locations were calculated by comparing the observed overlap frequency with the overlaps for 1000 random shuffling of the binding/differential binding peak locations [calculated using BEDTools 2.17.0 (*66*)].

### Calculation of regulatory targets of transcription factors

To identify likely targets of each transcription factor of interest, we drew upon the transcription factor binding site calculations described in (*28*) in which the motif of each transcription factor was scanned along every base pair of the *D. melanogaster* dm6 genome using FIMO (*71*) and the base-pairwise binding results were converted to robust *z* scores. For each TF, we then considered its regulon to consist of all genes with at least one binding site with *z* score above a TF-specific threshold within 2-kb upstream of the beginning of the gene. We identified TF-specific thresholds by manual inspection of a plot of the average expression changes between conditions versus threshold, aiming to identify a point of maximum information content relative to noise (see fig. S9 for the plots used to identify TF-specific *z* score thresholds). Once the set of targets (regulon) for each factor was identified, we tested for significant enrichment or depletion of overlaps between the regulons using Fisher’s exact test, reporting Benjamini-Hochberg FDRs (*70*). All calculated odds ratios were positive, indicating enriched overlaps between the regulons.

### STRING network analysis

To develop a functional network between the candidate neural targets of Cad, Ptx1, Nub, GATAe, and Scro, we uploaded genes categorized into neural/signaling GO terms based on DAVID functional annotations (*72*) to the STRING database (*34*), which includes both predicted physical and functional protein-protein interactions (*34*). Genes were clustered by their reported protein-protein interactions and corresponding confidence scores (*34*) and plotted in Cytoscape (v3.7.1). In this network, edges do not represent direct protein-protein interaction but rather represent a functional interaction. For network file, see file S2.

### Data analysis and statistics

Statistical tests, sample size, and *P* or *q* values are listed in each figure legend. For all PER experiments, Kruskal-Wallis Dunn’s multiple comparisons were used. Comparisons are either to control diets within genotypes or transgenic controls within dietary conditions. Data were evaluated for normality and appropriate statistical tests were applied if data were not normally distributed; all the tests, biological samples, and the *P* and *q* values are listed in the figure legends and specific analysis under each methods session. Because the inferential value of a failure to reject the null hypothesis in frequentist statistical approaches is limited, for all RNA-seq expression datasets, we coupled our standard differential expression with a test for whether each gene could be flagged as “significantly not different.” Defining a region of practical equivalence as a change of no more than 1.5-fold in either direction, we tested the null hypothesis of at least a 1.5-fold change for each gene, using the gene-wise estimates of the SE in log_2_fold change (reported by Deseq2) and the assumption that the actual l_2_fcs are normally distributed. Rejection of the null hypothesis in this test is taken as positive evidence that the gene’s expression is not changed substantially between the conditions of interest. Python code for the practical equivalence test can be found on GitHub as calc_sig_unchanged.py. All data in the figures are shown as means ± SEM, **** *P* < 0.0001, *** *P* < 0.001, ** *P* < 0.01, and *P < 0.05, unless otherwise indicated. Statistical analysis and tests are listed in every legend, unless otherwise noted in the text.

## Supplementary Material

http://advances.sciencemag.org/cgi/content/full/6/46/eabc8492/DC1

File S1

File S2

Adobe PDF - abc8492_SM.pdf

Persistent epigenetic reprogramming of sweet taste by diet

## SUPPLEMENTARY MATERIALS

Supplementary material for this article is available at http://advances.sciencemag.org/cgi/content/full/6/46/eabc8492/DC1

## Appendix

View/request a protocol for this paper from *Bio-protocol*.
